# Differences in metabolic profiles between bicuspid and tricuspid aortic stenosis in the setting of transcatheter aortic valve replacement

**DOI:** 10.1186/s12872-020-01491-4

**Published:** 2020-05-18

**Authors:** Tian-Yuan Xiong, Chang Liu, Yan-Biao Liao, Wen Zheng, Yi-Jian Li, Xi Li, Yuanweixiang Ou, Zi-Jie Wang, Xi Wang, Chang-Ming Li, Zhen-Gang Zhao, Yuan Feng, Xiao-Jing Liu, Mao Chen

**Affiliations:** 1 Department of Cardiology, West China Hospital, Sichuan University, #37 Guo Xue Alley, Chengdu, 610041 People’s Republic of China; 2 Laboratory of Mitochondrial Biology, West China-Washington Mitochondria and Metabolism Center, West China Hospital, Sichuan University, Chengdu, People’s Republic of China; 3 Laboratory of Cardiovascular Diseases, Regenerative Medicine Research Center, West China Hospital, Sichuan University, 610041 Chengdu, People’s Republic of China

**Keywords:** Bicuspid aortic valve, Aortic stenosis, Metabolomics, Transcatheter aortic valve replacement, Inflammation

## Abstract

**Background:**

To explore why bicuspid aortic stenosis has certain clinical differences from the tricuspid morphology, we evaluated the metabolomics profile involved in bicuspid aortic valve (BAV) aortic stenosis prior to and after transcatheter aortic valve replacement (TAVR) in comparison with tricuspid aortic valve (TAV).

**Methods:**

In this TAVR cohort with prospectively collected data, blood samples were obtained before TAVR valve deployment and at the 7th day after TAVR, which were then sent for liquid and gas chromatography-mass spectrometry detection. Besides comparisons between BAV and TAV, BAV patients were also divided in subgroups according to baseline hemodynamics (i.e. maximal transaortic velocity, V_max_) and post-procedural reverse left ventricular (LV) remodeling (i.e. the change in LV mass index from baseline, ∆LVMI) for further analysis. Metabolic differences between groups were identified by integrating univariate test, multivariate analysis and weighted correlation network analysis algorithm.

**Results:**

A total of 57 patients were enrolled including 33 BAV patients. The BAV group showed lower arginine and proline metabolism both before and post TAVR than TAV represented by decreased expression of L-Glutamine. In BAV subgroup analysis, patients with baseline V_max_ > 5 m/s (*n* = 11) or the 4^th^ quartile of change in ∆LVMI at one-year follow-up (i.e. poorly-recovered LV, *n* = 8) showed elevated arachidonic acid metabolism compared with V_max_ < 4.5 m/s (*n* = 12) or the 1^st^ quartile of ∆LVMI (i.e. well-recovered LV, *n* = 8) respectively.

**Conclusions:**

Difference in arginine and proline metabolism was identified between BAV and TAV in TAVR recipients. Elevated arachidonic acid metabolism may reflect more severe baseline hemodynamics and worse LV reserve remodeling after TAVR in BAV.

## Background

Bicuspid aortic valve (BAV) is the most common congenital defect characterized by two leaflets in the aortic position, which may have a spectrum of clinical presentations [1, 2]. In terms of aortic stenosis, some interesting distinctions have been observed in BAV patients. Compared with normally formed tricuspid aortic valve (TAV), BAV patients often develop more severe stenosis with heavier calcification and/or fibrosis in aortic leaflets at a younger age [3]. The difference is not limited to the valve itself, but also seems to involve the left ventricle (LV). Patients with BAV were reported to undergo less pronounced reverse LV remodeling than TAV [4] after definitive treatment by means of surgical or transcatheter aortic valve replacement (TAVR). Despite these observations indicating bicuspid aortic stenosis to have its own characteristics, current follow-up and treatment of bicuspid aortic stenosis do not differ from TAV patients.

A handful of studies have indeed identified some intrinsic genetic bases engaged in the etiology of BAV, such as the gene of ACTA2 [5] and NOTCH1 [6]. However, as the final stage in processing genomic information, metabolites were considered to significantly influence cellular activity and directly reflect extracellular microenvironment [7, 8]. Thus, metabolomics profiling technologies [9] are more likely to determine novel disease biomarkers and provide insights in biological mechanisms, which would be essential to improve treatment strategies. To date, few studies have focused on the specific metabolic mechanisms involved in bicuspid aortic stenosis, especially after aortic valve replacement.

In this prospective study, we sought to incorporate metabolomics profiling technologies in the setting of TAVR, with patients of both bicuspid and tricuspid morphology. In particular, detailed analyses are planned for BAV patients to explore potential metabolomic differences in subgroups with worse baseline hemodynamics and poorer LV recovery post-TAVR.

## Methods

### Study population

From November 2015 to August 2016, patients with severe symptomatic aortic stenosis, who successfully underwent TAVR in our center with both pre- and post-procedural blood samples collected, were enrolled in the study, while patients who received TAVR due to failed bioprostheses were excluded (*N* = 57, 32 females). Patients were divided into BAV and TAV groups according to their valve morphology confirmed and classified on multi-slice computed tomography (MSCT). TAVR was performed by the same heart team from the transfemoral access under general anesthesia, as described previously [10]. All enrolled patients received the same management process and were treated with the same intervening protocol. Participants enrolled in this study signed the informed consent in accordance with the Declaration of Helsinki. This study was approved by the Institutional Review Board of West China Hospital, Sichuan University.

### Echocardiography follow-up

Transthoracic echocardiography (TTE) were arranged before discharge and at routine follow-ups at the 1st month, 3rd month, 6^th^month and 1st year after TAVR. To assess reverse LV remodeling after TAVR, LV mass (LVM) and LVM index (LVMI) were calculated [11]. The change in LVM and LVMI was calculated as the value at follow-ups subtracted the value at baseline (shown as ∆LVM and ∆LVMI) and served as a surrogate endpoint to LV recovery post-TAVR.

### Blood sample collection and processing

Blood sample was obtained from peripheral vein before valve deployment during the index TAVR procedure and at the 7th day after TAVR (normally the day of discharge) in K2EDTA-treated tubes (BD). Subsequently, these blood samples were centrifuged at 2000 g for 10 min to pellet the cellular elements. The supernatant plasma was stored at − 80 °C until sample preparation for liquid chromatography-mass spectrometry (LC-MS) and gas chromatography-mass spectrometry (GC-MS) analysis.

### Metabolomics detection

Metabolomics profiling was detected and processed by Chinese Academy of Sciences in Dalian based on methods described in previous studies [12, 13]. Briefly, metabolomic analysis was conducted on ACQUITY Ultra Performance Liquid Chromatography (UPLC, Waters Corporation, Manchester, UK) system with ACQUITY UPLC BEH C8 1.7 μm (2.1 × 100 mm) column (Waters, Milford, MA) for positive ion metabolites separation setting at 50 °C and ACQUITY UPLC HSS T3 1.8 μm (2.1 × 100 mm) column (Waters, Milford, MA) for negative ion metabolites separation setting at 50 °C. A QP 2010 Plus GC-MS system (Shimadzu, Japan) with a DB-5MS (30 m × 250 μm × 0.25 μm, Agilent Technologies, USA) was used in GC-MS analysis. The procedures were performed in accordance with the manufacturers’ protocols of all devices. Then, raw data were converted to mzData formats via Agilent MassHunter Qualitative software (Agilent, Santa Clara, CA, USA). The program XCMS (version 1.40.0) (https://xcmsonline.scripps.edu/) was used to preprocess the raw data, with the default parameters. In the quality control (QC) process, nearly 100% features in QC samples had relative standard deviation (RSD) distribution less than 30%, and were within 3SD, which demonstrated a satisfactory data quality. The resulting matrix was constructed by retention time, mass-to-charge ratio (m/z), and normalized ion intensities.

### Metabolomics interpretation

The model of partial least squares discriminant analysis (PLS-DA) was used to classify metabolic characteristics of bicuspid aortic stenosis from tricuspid aortic stenosis. The variable importance project (VIP) from PLS-DA was also calculated. Furthermore, *p*-value from t-test analysis and fold-change value (FC) were executed to discover differentially expressed metabolites, which were exhibited by the volcano plot. With a pre-specified cutoff of *p*-value< 0.05, FC > 1.2 and VIP > 1.2, the dominant molecules were to be identified. These analyses were completed with an in-house script by R platform (https://www.r-project.org).

To futher identify the essential metabolic pathways and hub metabolites engaged in phenotypic alterations, pathway enrichment analysis was employed to summarize more interpretable results [14]. However, if the number of metabolites identified above was over 100, weighted gene co-expression network analysis (WGCNA) algorithm was to be first utilized to narrow down the number of metabolites sent to pathway enrichment analysis [15]. This protocol enabled the detection of hub molecules which were also correlated with clinical traits. Metabolites to be sent to patheway enrichment analysis were then selected by Venn diagram of both hub molecules defined by the WGCNA protocol and differentially expressed metabolites defined by the volcano plot. Pathway enrichment analysis was conducted with MetaboAnalyst platform (http://www.metaboanalyst.ca) [16].

### Subgroup analysis

For the subgroup analysis to explore the specific metabolites related to worse hemodynamics in BAV patients before TAVR, 4 m/s < maximal transaortic velocity (V_max_) < 4.5 m/s was defined as the lower V_max_ group, whereas V_max_ > 5 m/s was defined as the higher V_max_ group. Post-procedurally, the value of ∆LVMI at 1 year was divided into quartiles. BAV patients in the 4^th^ quartile were regarded to have the worst LV recovery, while those in the 1^st^ quartile were regarded to have the best LV recovery.

### Statistical analysis

Normality was assessed for all clinical datasets by the Shapiro-Wilk’s test. Continuous results were presented as the mean ± SD or median (IQR) according to their distribution. Independent t-test (for variables that were normally distributed) or Mann-Whitney U-test (for variables that were not normally distributed) was carried out to assess differences in clinical features between individuals in two groups. A two-tailed *p* value < 0.05 was considered as significant.

## Results

### Clinical features of study cohort

A total of 57 patients were enrolled with blood sample collected at the two time points. Baseline was summarized in Table 1. Confirmed with MSCT, there were 33 patients with BAV in this cohort. The average age was similar between BAV and TAV groups (72.48 ± 5.62 vs. 74.38 ± 5.53 years, *P* = 0.21). Two patients were low-flow, low-gradient aortic stenosis with reduced ejection fraction, while others were high-gradient aortic stenosis. BAV patients had higher V_max_ (5.14 ± 0.78 vs. 4.57 ± 0.85 m/s, *P* = 0.01) but lower LVM (265.35 [87.38] vs. 338.84 [98.46] g, *P* < 0.01) than TAV patients on pre-procedural TTE. Calcium volume of the aortic valve measured on MSCT was similar between BAV and TAV patients (705.70 [485.15] vs. 535.25 [598.93] m^3^, *P* = 0.27).

**Table 1:** Clinical features of enrolled patients

	Bicuspid aortic stenosis (*n* = 33)	Tricuspid aortic stenosis (*n* = 24)	*P*-value
Female, n (%)	24 (72.73%)	8 (33.33%)	0.006
Age (years)	72.48 ± 5.62	74.38 ± 5.53	0.21
Height (m)	1.57 ± 0.08	1.61 ± 0.09	0.12
Weight (kg)	55.15 ± 9.88	59.69 ± 9.87	0.92
BMI (kg/m^2^)	22.13 ± 3.45	22.83 ± 3.23	0.44
STS-PROM (%)	6.50 (4.42–8.59)	8.26 (4.93–13.38)	0.09
Comorbidities			
Hypertension, n(%)	15 (45.45%)	7 (29.17%)	0.27
Diabetes, n (%)	1 (3.03%)	3 (12.5%)	0.15
Chronic lung disease, n (%)	18 (54.55%)	19 (79.17%)	0.09
Coronary artery disease, n (%)	16 (48.48%)	10 (41.67%)	0.78
Prior myocardial infarction, n (%)	0	0	–
Prior percutaneous coronary intervention, n (%)	2 (6.06%)	3 (12.5%)	0.64
Peripheral vascular disease, n (%)	20 (60.60%)	20 (83.3%)	0.08
Chronic kidney disease, n (%)	6 (18.18%)	5 (20.83%)	0.99
Baseline echocardiography			
Left ventricular ejection fraction, %	53.73 ± 15.05	44.67 ± 14.19	0.025
Transaortic V_max_, m/s	5.14 ± 0.78	4.57 ± 0.85	0.011
Transaortic PG_mean_, mmHg	67.52 ± 21.99	55.79 ± 16.01	0.031
LVM, g	265.35 ± 87.38	338.84 ± 98.46	0.004
LVMI, g/m^2^	161.66 ± 48.78	197.66 ± 62.32	0.018
Ascending aorta diameter, mm	40.50 ± 5.59	38.75 ± 4.32	0.21

Hemodynamics and reverse LV remodeling post-TAVR were illustrated in Supplementary Figure 1. The value of post-procedural V_max_, LVM and LVMI were comparable between the two groups through one-year follow-up, but BAV patients experienced less pronounced reverse LV remodeling than TAV patients (∆LVMI, − 35.35 ± 42.07 vs. -68.4 ± 56.89 g/m^2^, *P* = 0.03).

### Distinctions of arginine and proline metabolism pathway between patients with BAV and TAV

**Fig. 1:**
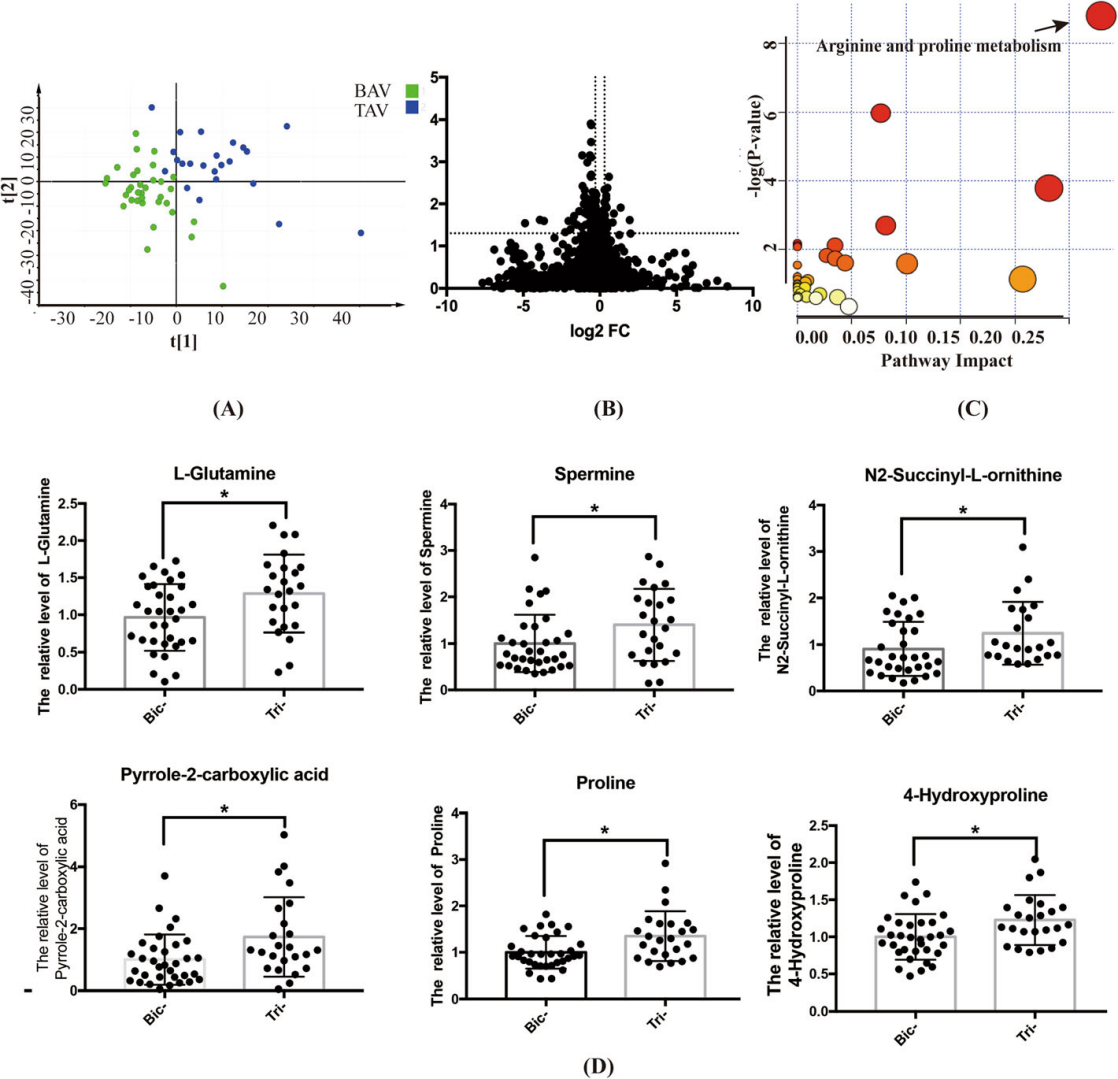
The specific metabolites differentially expressed between BAV and TAV patients prior to TAVR. **a** PLS-DA analysis implied the diverse metabolic patterns between bicuspid aortic stenosis and tricuspid aortic stenosis. **b** Comparison of all metabolites between BAV and TAV by volcano plot. **c** Enrichment analysis for these dominant metabolites which separated BAV stenosis participants from TAV stenosis ones (*p*-value< 0.05). **d** The expression of metabolites in arginine and proline metabolism pathway between BAV and TAV patients before TAVR (* indicated *p*-value< 0.05)

**Fig. 2:**
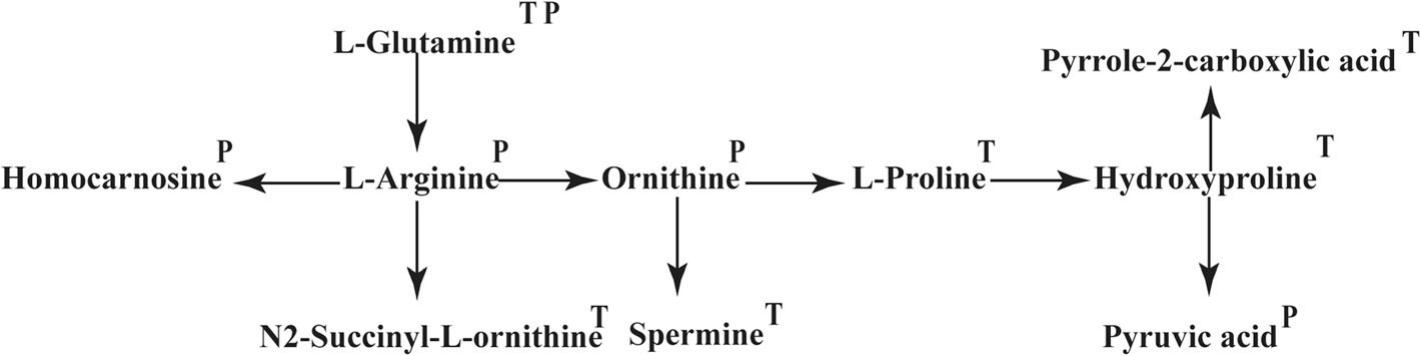
The metabolic map of arginine and proline metabolism pathway. Letter T indicated the elevated metabolites in TAV patients before TAVR compared with BAV; letter P indicated the elevated metabolites in TAV patients post-TAVR compared with BAV

Prior to TAVR, a diverse metabolic pattern of BAV from TAV was observed through the separation on PLS-DA score plot (Fig. 1a). By global metabolomics profiling, the differentially expressed metabolites of BAV from TAV were further identified with the cutoff of *P*-value < 0.05, FC-value> 1.2 and VIP value> 1.2 (Fig. 1b). According to the enrichment analysis, obviously altered metabolites were mapped to the arginine and proline metabolism pathway (Fig. 1c). In details, the expression of 6 metabolites in this pathway, namely L-Glutamine, L-Proline, Hydroxyproline, Pyrrole-2-carboxylic acid, N2-Succinyl-L-ornithine and spermine, was significantly lower in BAV than TAV (Fig. 1d). The locations of these remarkably altered molecules were further elucidated in the metabolic map of the arginine and proline metabolism pathway and marked with the letter T in Fig. 2.

**Fig. 3:**
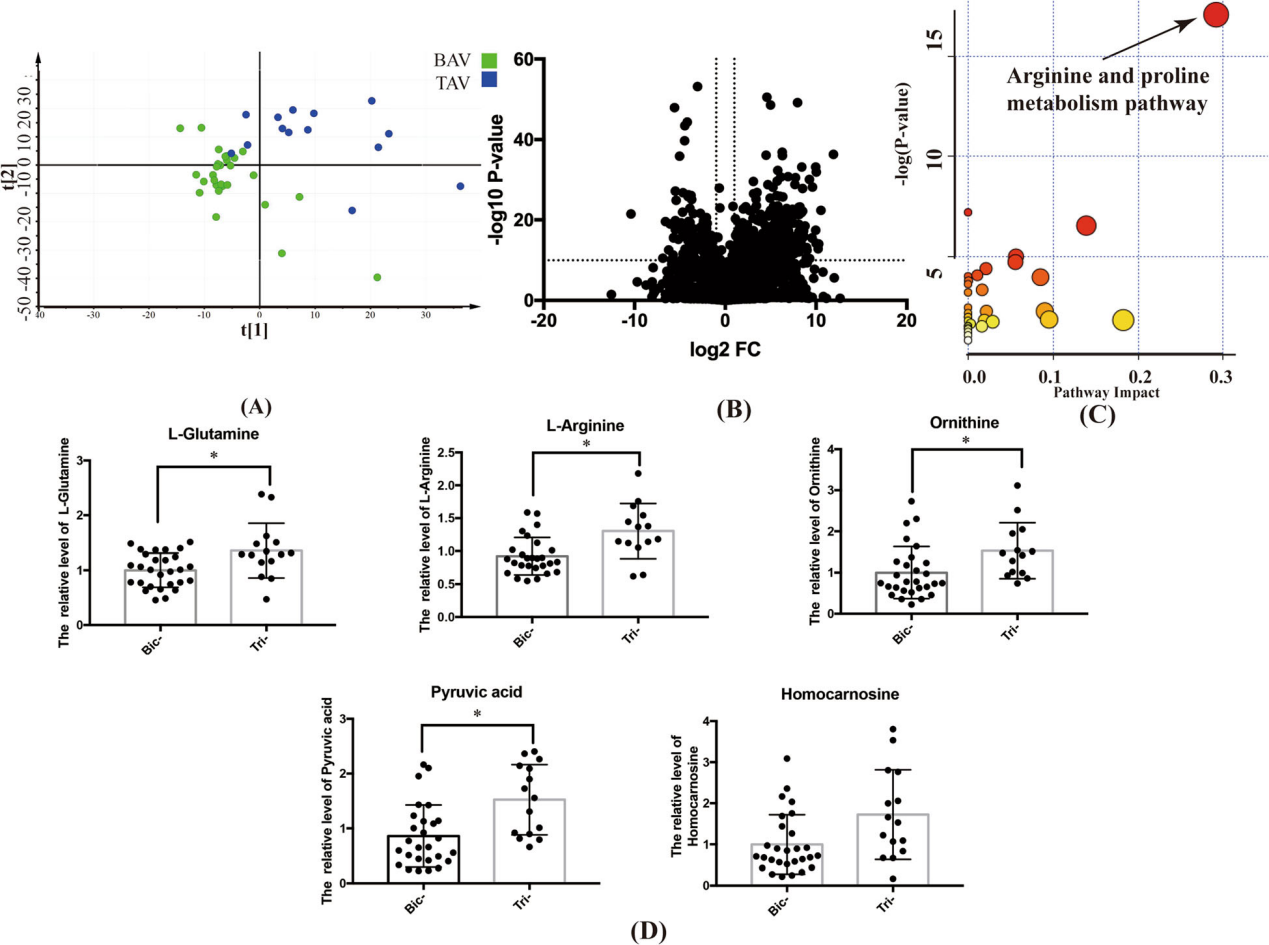
The specific metabolites differentially expressed between BAV and TAV patients 7 days after TAVR. **a** Metabolic profiling distinguished patients with bicuspid and tricuspid aortic stenosis after TAVR by PLS-DA. **b** Comparison of all metabolites between BAV and TAV by volcano plot. **c** The pathway enrichment analysis demonstrated obviously altered arginine and proline metabolism pathway for BAV patients after TAVR, comparing with TAV patients (*P*-value< 0.05). **d** The reduced metabolites of L-Glutamine, L-Arginine, Pyruvic acid, Homocarnosine and Ornithine in arginine and proline metabolism pathway in BAV patients (* indicated *P*-value< 0.05)

Seven days post-TAVR, the metabolic pattern still separated between BAV and TAV groups on PLS-DA model (Fig. 3a). With the same cutoff of *P*-value, FC and VIP, differentially expressed molecules between the two groups were identified and shown by the volcano plot (Fig. 3b). On the basis of enrichment analysis, these identified metabolites were mapped, again, to the arginine and proline metabolism pathway (Fig. 3c). The expression of 5 metabolites in this pathway, namely L-Glutamine, L-Arginine, Pyruvic acid, Homocarnosine and Ornithine, was significantly lower in BAV than TAV (Fig. 3d). In the metabolic map of the arginine and proline metabolism pathway, they were marked with the letter P in Fig. 2.

### Subgroup analysis of BAV

**Fig. 4:**
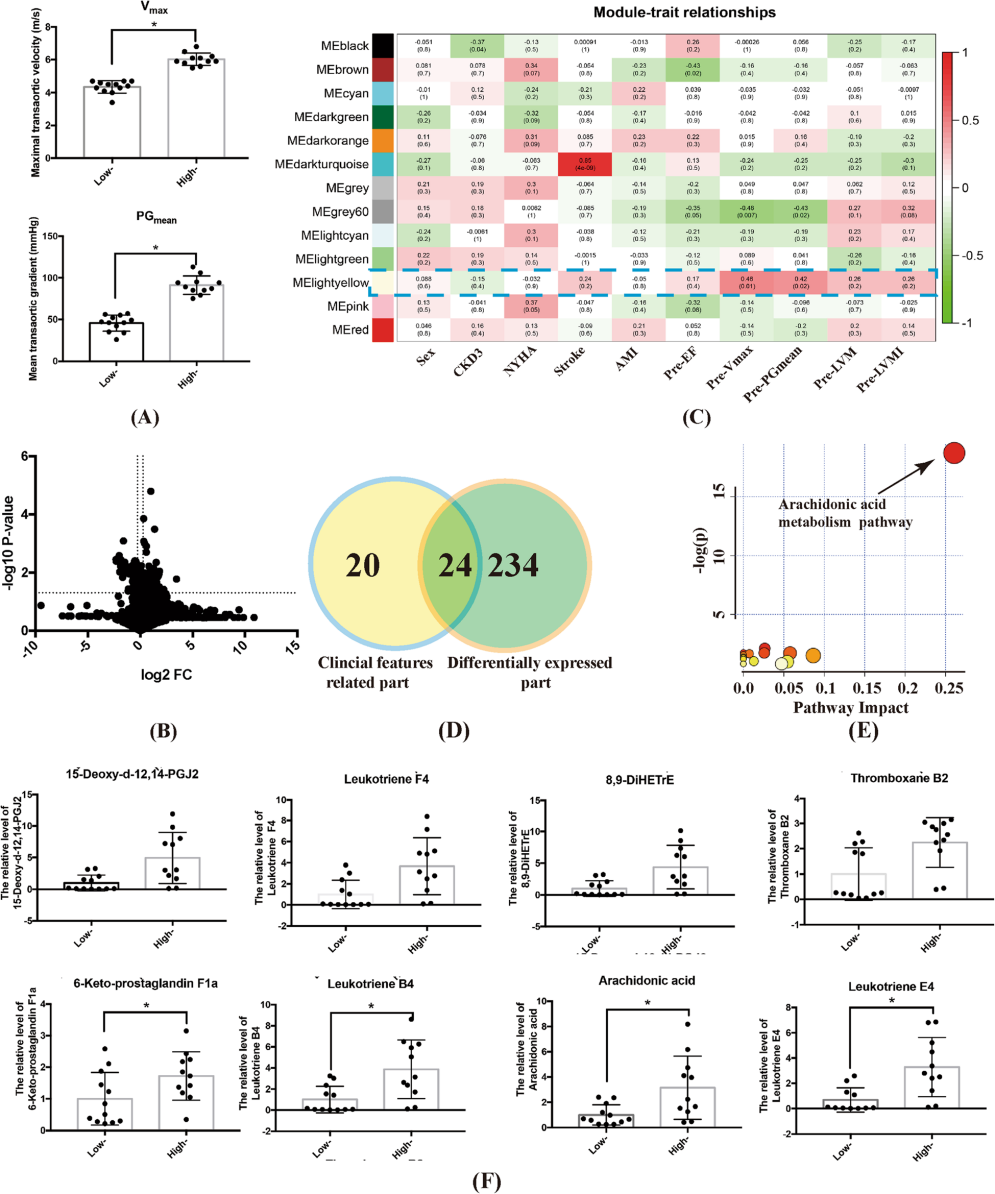
Baseline subgroup analysis for BAV patients. **a** Baseline hemodynamics in lower and higher baseline V_max_ groups. (* indicated *p*-value< 0.05). **b** Comparison of all metabolites between the lower and higher V_max_ groups by volcano plot. **c** WGCNA algorithm identified the metabolic module closely related to baseline V_max_. **d** Venn plot indicated the impact metabolites which both were positively related to baseline V_max_ and differentially expressed between BAV and TAV. **e** Pathway enrichment analysis detected the significantly altered arachidonic acid metabolism pathway (*p*-value< 0.05). **f** The different expression of metabolites in arachidonic acid metabolism pathway between patients with lower and higher baseline V_max_ (* indicated *p*-value< 0.05)

**Fig. 5:**
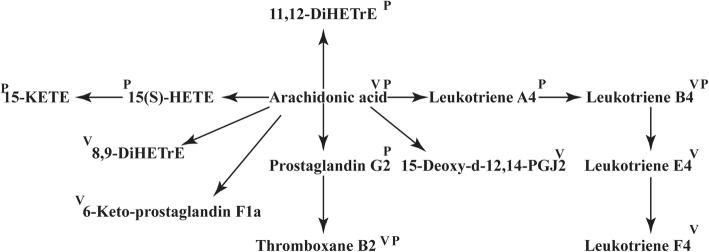
The metabolic map of arachidonic acid metabolism pathway. The metabolites elevated in BAV patients with higher baseline Vmax were labeled by letter V. Those elevated in BAV patients with ∆LVMI value within the 1^st^ quartile were labeled by letter P

At baseline, 12 BAV patients were identified as the lower (4 m/s < V_max_ < 4.5 m/s) and 11 as the higher (V_max_ > 5 m/s) V_max_ group respectively. Details were shown in Fig. 4a. By univariate and multivariate analysis, we identified 258 differentially expressed metabolites between these two groups (Fig. 4b). WGCNA algorithm revealed hub molecules correlated with pre-procedural V_max_ and PG_mean_, i.e. the MElightyellow module (labeled in blue frame) (Fig. 4c). A total of 24 hub metabolites were determined (Fig. 4d) and then mapped to the arachidonic acid metabolism pathway by enrichment analysis (Fig. 4e). In details, 8 metabolites including 6-Keto-prostaglandin F1a, Leukotriene B4, Arachidonic acid, Leukotriene E4, and etc., expressed significantly less in the lower than higher V_max_ group (Fig. 4f). The location of these molecules was listed in the metabolic map of the arachidonic acid metabolism pathway, which were labeled by letter V in Fig. 5.

**Fig. 6:**
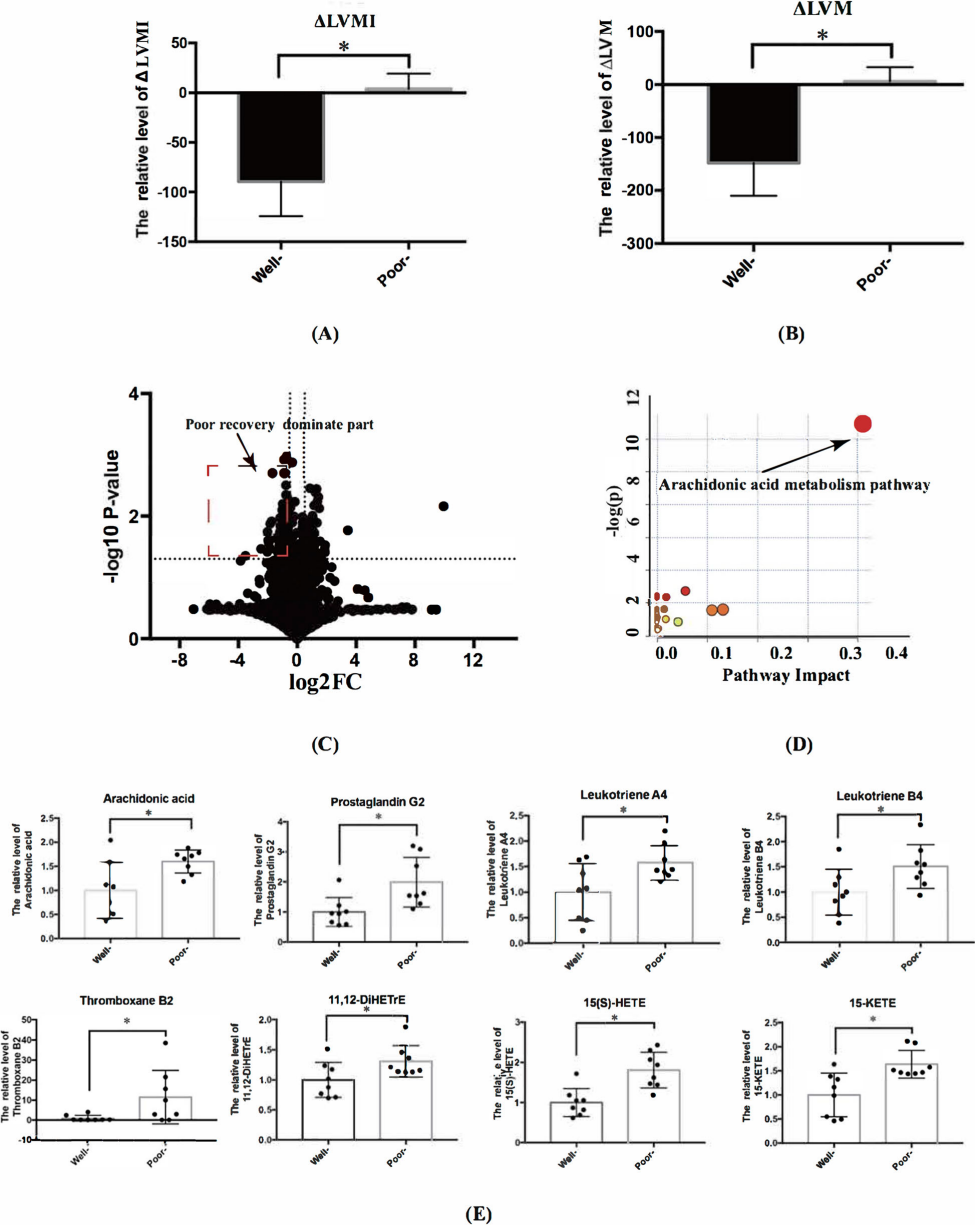
Post-procedural subgroup analysis for BAV patients. **a** One-year ∆LVMI and (**b**) ∆LVM in the 1^st^ and 4^th^ quartile groups (all *P*-value< 0.05). **c** Volcano plot showed the differentially expressed metabolites in the two groups. **d** The pathway enrichment analysis demonstrated elevated arachidonic acid metabolism pathway in patients within the 1^st^ quartile of ∆LVMI (*P*-value< 0.05). **e** The different expression of metabolites in arachidonic acid metabolism pathway between patients within the 1^st^ and 4^th^ quartile of one-year ∆LVMI (* indicated *P*-value< 0.05)

At one-year follow-up, 8 patients within the 4^th^ quartile of ∆LVMI value were identified as the poorly-recovered group and 8 patients within the 1^st^ quartile of ∆LVMI value as the well-recovered group (Fig. 6a and b). By vocalno plot, 37 differentially expressed metabolites in these two groups negatively related to ∆LVMI were detected (Fig. 6c) and mapped, again, to the arachidonic acid metabolism pathway (Fig. 6d). The metabolites of 15-KETE, 15(S)-HETE, arachidonic acid, prostaglandin G2, Thromboxane B2, Leukotriene A4 and Leukotriene B4 were significantly increased in patients in the poorly-recovered group (Fig. 6e). Their locations were listed in in Fig. 5.

## Discussion

The incorporation of metabolomics profiling technologies to TAVR patients offered us a unique way to explore bicuspid aortic stenosis in comparison with the normal tricuspid morphology, both before and after the stenotic status been corrected. The two major findings of this study are (1) BAV patients had an altered arginine and proline metabolism pathway compared with TAV, which sustained post-TAVR; (2) the arachidonic acid metabolism pathway seems to be associated with more severely affected hemodynamics, as well as poorly-recovered LV post-TAVR in BAV. These preliminary results provide novel insights to further elucidate the different disease spectrum of bicuspid aortic stenosis and determine possible biomarkers for monitoring progression and prognosis of bicuspid aortic stenosis.

Patients with bicuspid aortic stenosis are known to require aortic valve replacement at a much younger age than their tricuspid counterparts, usually within productive years of life [17]. As we currently do not have means to effectively postpone the disease progress of aortic stenosis, determining the difference between bicuspid and tricuspid aortic stenosis may be an alternative way for BAV patients to at least face this inevitable intervention later in their life. Elevated arginine and proline metabolism pathway was found in TAV compared with BAV, regardless whether severe aortic stenosis was corrected or not. This finding suggested that metabolites in the arginine and proline pathway may be potential targets to delay the progression of bicuspid aortic stenosis. The arginine and proline metabolism pathway generates nitric oxide with Nitric Oxide Synthase (NOS) [18, 19]. Elevated endothelial nitric oxide synthase (eNOS) has been found in TAV [20], but eNOS was low expressed and irregularly distributed in BAV [21]. ENOS is essential in regulating biological homeostasis. Limited eNOS reduces cellular NO level which could lead to oxidative stress, and promote oxidative damage [22, 23]. Besides key enzymes in arginine and proline metabolism pathway, the detected crucial amino acids such as glutamine and proline, likewise, have biological significance for bicuspid aortic stenosis. Proline was previously reported to be the balancer of over oxidation, which was engaged in stabilizing proteins and antioxidant enzymes, and directly scavenging ROS [24]. A study has also demonstrated that glutamine effectively controlled systemical inflammatory reactions, especially the molecules of IL-1, IL-6, IL-8, and IL-10 [25], which might be the proposed factors for accelerating valve stenosis [26]. Thus, targeting eNOS or the key amino acids in arginine and proline metabolism pathway might be potential targets to delay the progression of bicuspid aortic stenosis. Previously, perturbations in fatty acid metabolism has been identified in calcific coronary artery disease stratified by the severity of calcification [27, 28]. As a pathology with some similar characteristics, calcific aortic valve disease might share these alterations. However, in this analysis, BAV patients did not achieve a statistically higher calcium burden on the leaflets than TAV patients and the coronary calcium score was not assessed, making it difficult to evaluate the impact of total calcium burden on metabolomic profiling.

In the BAV subgroup analysis, both more severely affected baseline hemodynamics and poorly recovered LV post-TAVR were linked to elevated arachidonic acid metabolism pathway. As an important pathway for inflammation, the alleviative arachidonic acid metabolism might be crucial to halt the pathologic progression of aortic stenosis, especially for BAV patients [29, 30]. Within this pathway, the key enzyme of COX-2 was considered as an important drive for aortic stenosis by increasing valvular calcification and enhancing osteogenic genes including OPN and Runx2 [29]. In addition, COX-2 was reported to be central in the AngII-induced macrophage recruitment and the expression of TNF-α of endothelial cells, which blockades macrophage infiltration and exaggerates expression of pro-inflammatory cytokines [30]. Although there was no research reporting the role of arachidonic acid metabolism pathway in the recovery phase after aortic valve replacement, it was otherwise demonstrated to influence myocardial functions. Targeting metabolites such as cyclooxygenases and leukotrienes B4 in the arachidonic acid metabolism pathway protected against heart failure, decreased myocardial fibrosis and rescued myocardial function [31, 32]. The involved mechanisms might lie in preserving microcirculation and limiting inflammation, such as inhibiting TNF-α-mediated pathway and inflammatory mediators [33, 34]. Thus, targeting arachidonic acid metabolism pathway might be of tremendous therapeutic value in clinical practice and merits further detailed researches.

There were several limitations in this study. Firstly, blood samples were sent for metabolomics detection without particular filtering. Secondly, there was gender imbalance possibly by coincidence at the time of this study, but cautions should be taken when interpreting the difference of metabolomic profiling between the two valve morphologies due to this selection bias. Thirdly, it was difficult to draw definitive conclusions due to relatively small sample size without a validation cohort and limited related researches in this area. A larger metabolomics study combining validation experiments should be arranged to verify our findings and explore the detailed mechanisms of molecules involved in bicuspid aortic stenosis.

## Conclusions

This study comprehensively conducted metabolomics profiling for bicuspid aortic stenosis in the setting of TAVR, in comparison with TAV. Essential molecules in the arginine and proline metabolism pathway might delay the fast progression of bicuspid aortic stenosis. Metabolites in the arachidonic acid metabolism pathway might be potential biomarkers to dertermine BAV patients who would suffer from worse baseline hemodynamic stress or compromised LV recovery post-TAVR. Further investigations are needed to validate these findings and explore underlying biological functions for these influential metabolic pathways.

## Supplementary information

**Additional file 1.** The clinical features and follow-up data for enrolled patients (A) maximal transaortic velocity (V_max_); (B) mean transaortic gradient (PG _mean_); (C) left ventricular mass (LVM); (D ) left ventricular mass index (LVMI); (E) left ventricular ejection fraction (EF); (F) ΔLVM= left ventricular mass (LVM; 1 year after TAVR) – LVM (before TAVR); (G) ΔLVMI = left ventricular mass index (LVMI, 1 year after TAVR)– LVMI (before TAVR); (* indicated *P*-value< 0.05).

## Data Availability

The data are available from the corresponding author on reasonable request.

## Abbreviations

BAV: Bicuspid aortic valve
TAV: Tricuspid aortic valve
LV: Left ventricle
TAVR: Transcatheter aortic valve replacement
MSCT: Multi-slice computed tomography
TTE: Transthoracic echocardiography
LVM: LV mass (LVM)
LVMI: LV mass index
LC-MS: Liquid chromatography-mass spectrometry
GC-MS: Gas chromatography-mass spectrometry
QC: Quality control
SD: Standard deviation
PLS-DA: Partial least squares discriminant analysis
VIP: Variable importance project
FC: Fold change
WGCNA: Weighted correlation network analysis
NOS: Nitric oxide synthase
