# Supplementary figures and images for: Differences in metabolic profiles between bicuspid and tricuspid aortic stenosis in the setting of transcatheter aortic valve replacement
Source: BMC Cardiovasc Disord. 2020 May 18;20:229. doi: 10.1186/s12872-020-01491-4 (PMC7236099; doi:10.1186/s12872-020-01491-4)

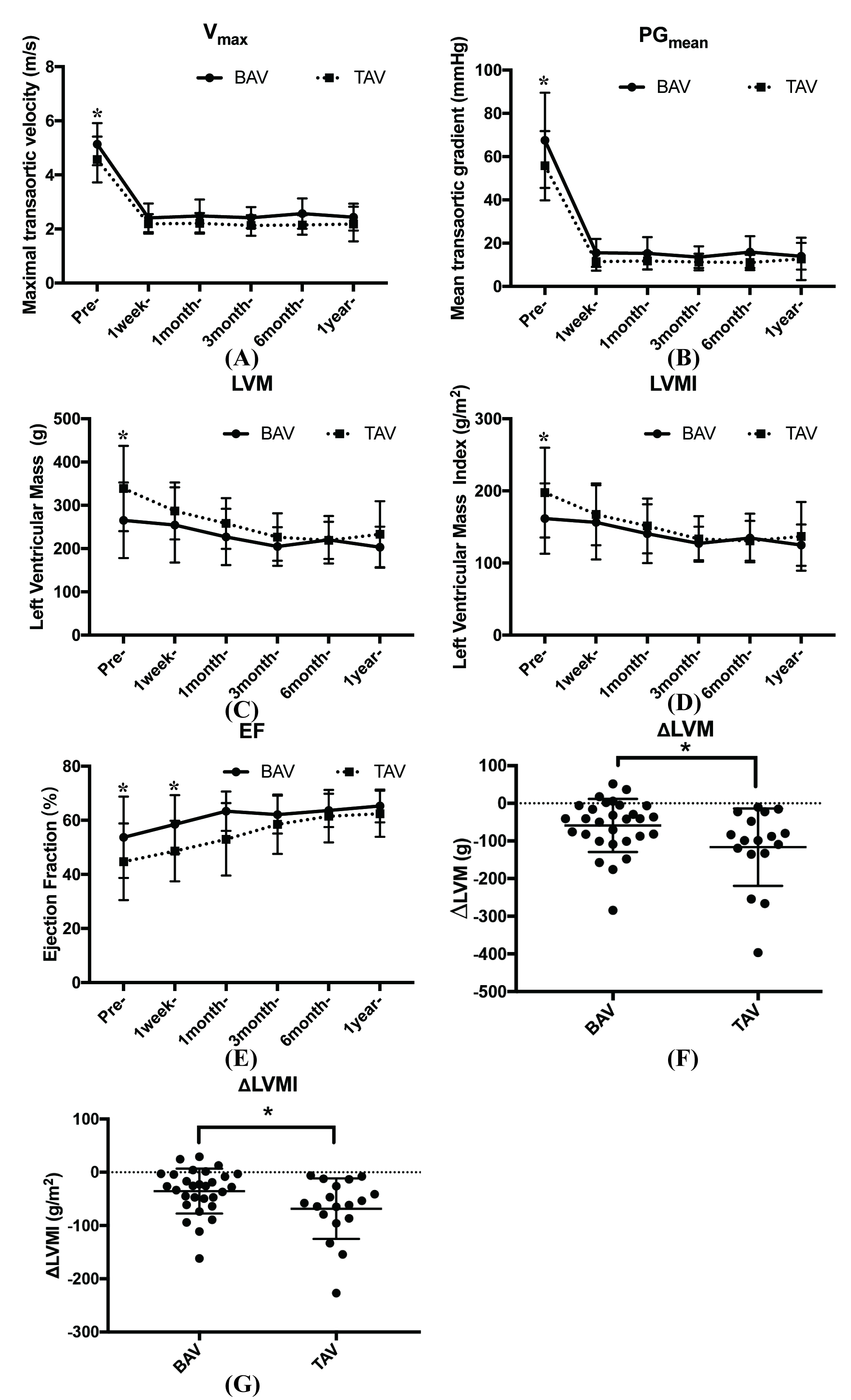

Supplement: Supplementary file 1 — Additional file 1. The clinical features and follow-up data for enrolled patients (A) maximal transaortic velocity (Vmax); (B) mean transaortic gradient (PG mean); (C) left ventricular mass (LVM); (D ) left ventricular mass index (LVMI); (E) left ventricular ejection fraction (EF); (F) ΔLVM= left ventricular mass (LVM; 1 year after TAVR) – LVM (before TAVR); (G) ΔLVMI = left ventricular mass index (LVMI, 1 year after TAVR)– LVMI (before TAVR); (* indicated P-value< 0.05). [file 12872_2020_1491_MOESM1_ESM.tif]
